# The Fascinating Cross-Paths of Pathogenic Bacteria, Human and Animal Faecal Sources in Water-Stressed Communities of Vhembe District, South Africa

**DOI:** 10.3390/pathogens12091085

**Published:** 2023-08-26

**Authors:** Mulalo Mudau, Renay Ngobeni-Nyambi, Maggy Ndombo Benteke Momba

**Affiliations:** 1 Department of Environmental, Water and Earth Sciences, Tshwane University of Technology, Arcadia Campus, Private Bag X680, Pretoria 0001, South Africa; 2 Department of Medicine, Division of Infectious Diseases, University of North Carolina School of Medicine, Chapel Hill, NC 27599, USA; renayngobeni@gmail.com

**Keywords:** enteric pathogens, faecal pollution, water-stressed areas, multiplex real-time PCR, rural areas

## Abstract

Access to clean and safe drinking water still remains a major challenge in the developing world, causing public health risks in terms of waterborne infections, especially in rural areas of sub-Saharan Africa. This study aimed to track and detect enteric pathogens (*Salmonella enterica* subsp. *enterica* serovar Typhimurium str. LT2, *Shigella flexneri*, and *Campylobacter jejuni* subsp. *jejuni*) in rural water sources. It also sought to establish a correlation between these pathogens and the sources of faecal pollution. Multiplex qPCR and specific primers and probes were used for detection and tracking. The study successfully correlated the occurrence of target pathogens with sources of human and animal faecal contamination using host-specific genetic markers (*BacHum* and *HF183* for humans, *BacCow* for cows, *Pig-2-Bac* for pigs, *Cytb* for chickens, and *BacCan* for dogs). The study revealed that enteric pathogens were found in 47.69% and 32.80% of samples during the wet and dry seasons, respectively. These pathogens were associated with human or animal faecal contamination. Correlations between pathogens and contamination sources were significant (*p* ≤ 0.05), with varying strengths during the wet and dry seasons. The findings emphasize the importance of identifying faecal contamination sources to protect rural communities from waterborne infections.

## 1. Introduction

Waterborne infections pose significant public health and regulatory concerns worldwide, affecting various water sources such as drinking water, wastewater, rivers, and lakes [1]. With microorganisms present in food, water, soil, and human and animal bodies, access to safe drinking water remains a challenge for many, leading to waterborne diseases and related deaths. Globally, about 525,000 children die from diarrhoeal diseases each year, and 2.5 billion people worldwide lack access to safely managed sanitation [2]. Estimates state that contaminated drinking water, poor sanitation, and inadequate hygiene contribute to 829,000 deaths in middle- and low-income countries annually [3]. Waterborne diseases have also been linked to more than 2.2 million annual deaths, making them a significant global health issue [4]. An estimated 66% of people worldwide contract waterborne and enteric diseases each year, including acute gastroenteritis, cholera, dysentery, hepatitis-A, and typhoid [5]. Consequently, 2.11 million South Africans are living in rural areas without safe water infrastructure, exposing them to waterborne illnesses and their complications [6]. Campylobacteriosis, shigellosis, salmonellosis, cholera, yersiniosis, and a number of other bacterial, fungal, viral, and parasitic infections are among the water-related diseases affecting people in South African rural communities [7].

Waterborne infections have severe effects on rural communities, exacerbating already burdensome healthcare needs and infrastructure challenges [5]. Salmonellosis, a major concern, causes millions of morbidity cases and over a million fatalities worldwide annually, characterized by fever, diarrhoea, abdominal pain, nausea, and vomiting [5]. In some regions, typhoidal *Salmonella* isolates lead to gastrointestinal illnesses with approximately 21.6 million positive cases and 200,000 fatalities per year [5]. *Shigella*, commonly found in human and primate intestinal tracts, is heavily discharged in faeces, persisting in water for extended periods and facilitating waterborne transmission [8]. There are an estimated 164.7 million *Shigella* episodes worldwide each year, with 163.2 million cases in underdeveloped countries causing 1.1 million fatalities, predominantly in children under five [8]. Symptoms like fever, anorexia, fatigue, and malaise are common with *Shigella* spp. [9]. *Campylobacter* is a prevalent bacterial cause of human gastroenteritis globally [10]. In 2008, nearly 200,000 confirmed cases of campylobacteriosis were reported, with *Campylobacter jejuni* being the primary cause of zoonotic disease in humans [5]. In many rural areas, dependence on private water wells and natural sources like rivers is necessary due to the lack of public water supplies and sewerage networks. Understanding contaminant sources and attenuation processes is essential for treating and disposing of domestic wastewater, ensuring safe drinking water in spatially constrained rural households [11]. The lack of access to safe water supply hinders efforts to promote good health, income, living conditions, equitable resource use, and a better quality of life in South Africa and other developing countries.

Like the majority of rural communities in developing countries, Vhembe District Municipality is facing challenges to supply clean water and improved sanitation to all; currently, those who lack improved water sources have to rely on highly contaminated sources. The Luvuvhu River serves as the primary source of water for the majority of the villages in the Vhembe District Municipality, either directly or through water treatment facilities. Previous investigators have reported that the river water of the Vhembe district has poor microbiological quality and is unfit for human consumption [7]. Due to its dense network of tributaries and extensive route through numerous villages, the Luvuvhu River is more vulnerable to contamination from various sources and it is extremely susceptible to waterborne pathogens. Therefore, the aim of the current study was to detect and track enteric pathogens (*Shigella flexneri*, *Salmonella enterica* subsp. *enterica* serovar Typhimurium str. LT2, and *Campylobacter jejuni* subsp. *jejuni*) from different water sources, which might contribute to outbreaks of water-related diseases in water-stressed rural communities. To achieve the set aim, multiplex real-time PCR using specific primers and probes was employed for the detection and tracking of enteric pathogens in different water sources of participating households. A correlation was then established between the occurrence of the target pathogens and the sources of human and animal faecal contamination in water sources.

## 2. Materials and Methods

### 2.1. Study Site Description, Water Supply, and Storage

The Vhembe District Municipality is situated in the northern part of the Limpopo Province of South Africa. It shares borders with the eastern district of Mopani and the western district of Capricorn. In 2016, a Stats SA [12] community survey revealed that the population of the Vhembe District was approximately 1,393,948, occupying a land area of 27,969,148 km^2^. This district is comprised of the local municipalities of Collins Chabane (CC), Thulamela (TM), Makhado (MK), and Musina (MS), with populations of 347.974, 497.237, 416.728, and 132.009, respectively. For this particular study, three local municipalities (Thulamela, Makhado, and Collins Chabane) were selected, and out of these, five villages experiencing water scarcity were randomly selected. These five villages were identified as Lambani, Tshifudi, Njhakanjhaka (Watervaal), Makuleke, and Gandlanani [13].

Within the Collins Chabane (CC) local municipality, the study focused on two villages, identified as Gandlanani and Makuleke. Gandlanani village receives its water supply from the Gandlanani Water Treatment Works, situated in the village, which draws its raw water from the Luvuvhu River, flowing through the village. On the other hand, Makuleke village relies on two treatment plants for its water supply, Xikundu Water Treatment Works and the Mhinga Water Treatment Works, depending on their operational status. The Xikundu Water Treatment Works, located in Xikundu village, draws its raw water from the Luvuvhu River passing through that area, while the Mhinga Water Treatment Works, situated in Mhinga village, also draws water from the Luvuvhu River.

**Figure 1:**
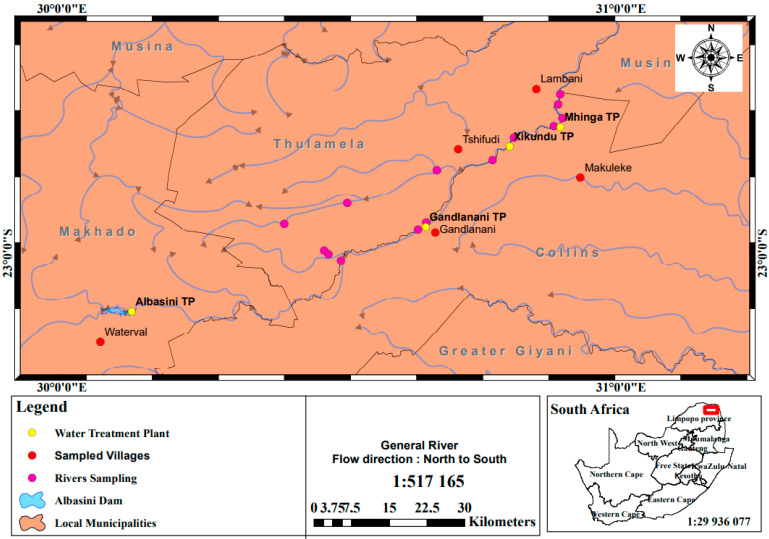
Map showing sampled villages, water treatment plants, and rivers.

In the Thulamela (TM) Local Municipality, the study included two villages, Tshifudi and Lambani, both of which receive their water supply from the Xikundu Water Treatment Works located in Xikundu village. Lastly, in the Makhado (MK) Local Municipality, one village, Njhakanjhaka, was selected, whose primary water supply is obtained from a treatment plant, Albasini Water Works, that draws water from the Albasini Dam. However, during the study period, the participating households in this village did not receive their water supply from this plant due to water shortages and blockage in the pipeline, leading them to purchase water from households with private boreholes in their vicinity. Figure 1 illustrates the selected villages, the water treatment plants providing water to these villages, and the rivers from which the treatment plants draw their water.

### 2.2. Ethical Clearance

Ethical clearance approval was granted by the Faculty of Science Research Ethics Committee at the Tshwane University of Technology (TUT). Permission was obtained from the Vhembe District Municipality and the community leaders to conduct the study in selected villages of this district after consultation and briefing them on the aim and objectives of the study. Informed consent was sought from community participants and plant managers after clearly explaining the motives behind the study and their involvement. Before taking part in the study, participants were asked to sign a consent form.

### 2.3. Sample Collection at Different Points

Water samples were collected sequentially from the main source all the way to the point of use: the storage containers in the households of the selected villages. Although the majority of households had standpipes in their yards, most of the villages did not have running water in their taps during the sampling periods. Thus, the sampling regime also included container-stored water in households. In villages where running water was available, water samples were collected from both the tap and the storage container. Water samples were collected eight (8) times at each sampling location. During the dry season, water samples were obtained four times at each sampling location, and similarly during the wet season. In total, 1.128 water samples were collected from the five communities as shown in Table 1. Seasonal water sampling was carried out at several water sources using known techniques and APHA 2001 plate count methods [14]. Water samples were transported on ice in cooler boxes to the water research laboratory at Tshwane University of Technology. Analysis was performed within 24 h after collection. 

### 2.4. Sample Concentration of Bacteria and DNA Extraction

Within 24 h of collection, all water samples (each measuring 500 mL) underwent filtration using a vacuum manifold. Specific filters were used for this process: mixed cellulose ester filters with a diameter of 47 mm and a pore size of 0.22 μm (obtained from Merck Millipore in Billerica, MA, USA and Merck SA, Singapore). However, in the case of river water samples, a larger filter with a diameter of 90 mm and a pore size of 0.45 μm (Merck Millipore) was used, following the method described by Haramoto and Osado [15].

To ensure that the samples were not contaminated during processing, sterile deionised water was filtered alongside them. The filter papers were then rolled and placed inside 5 mL PBS tubes. Each PBS tube received a drop of Tween^®^ 20 and was vortexed vigorously to dislodge any materials retained on the filter paper. Subsequently, the water samples underwent centrifugation at a high speed of 15,000 rpm for 10 min. After centrifugation, DNA extraction was performed on the resulting pellet using the Zymo Research DNA isolation kit (Quick-DNA Fecal/Soil Microbe Miniprep Kit, Sigma-Aldrich, St. Louis, MO, USA) following the instructions provided by the manufacturer. To account for potential contamination during DNA extraction, negative controls, known as blanks, were included in each extraction batch. The extracted genomic DNA was then stored in a freezer at −20 °C until further use.

### 2.5. Multiplex Real-Time PCR for the Detection of Enteric Pathogens

The DNA extracted from the water samples was subjected to multiplex real-time PCR for the detection of *Campylobacter jejuni* subsp. *jejuni*, *Shigella flexneri*, and *Salmonella enterica* subsp. *enterica* serovar Typhimurium str. LT2. The three different primer/probe sets used for the purpose of this study were previously developed by Wiemer et al. [16] to target the tetrathionate reductase subunit C (*ttrC*) gene for *Salmonella enterica* subsp. *enterica* serovar Typhimurium str. LT2, the DNA gyrase subunit A gene (*GyrA*) for *Campylobacter jejuni* subsp. *jejuni* and *Campylobacter jejuni* subsp. *doylei*, and finally the invasion plasmid antigen H (*ipaH*) gene for *Shigella flexneri*. Following the method used by Wiemer et al. [16] with slight modifications, the multiplex quantitative PCR (qPCR) assay was performed using CFX96 Touch Real-Time PCR Detection System combined with the C1000™ Thermal Cycler (Bio-Rad Laboratories, Hercules, CA, USA) and assays for detecting the above-mentioned pathogens. Table 2 lists the names and sequences of primers, probes, and cycling conditions used for the assay. 

The multiplex qPCR total reaction volume of 25 μL contained 12.5 μL of the GoTaq^®^ probe qPCR master mix, 7.5 μL of the primer/probe mix, 3 μL of nuclease-free water, and 2 μL of genomic DNA. Pure extracts of reference strains of the target pathogens were used (*Shigella flexneri* (serotype 2b) ATCC 12022, *Campylobacter jejuni* subsp. *jejuni* ATCC 33291, *Salmonella* Typhimurium ATCC 13311) as positive controls and nuclease-free water was used as a negative control together with DNA extraction blanks. 

For a standard curve, 10-fold serial dilution (10×) of plasmid DNA containing the target region to be amplified was used to prepare standards. The lowest cycle threshold (Ct) value obtained from the serially diluted samples was regarded as the limit of detection and a cut-off value for each assay was determined by the highest Ct value obtained during the optimisation run. Microbial source tracking markers were used to identify the possible sources of faecal contamination in water. As described in a study by Mudau [13], host-specific marker assays were used in real-time PCR for the identification of the possible sources of contamination. 

### 2.6. The Occurrence of Sources of Faecal Contamination in Different Water Sources

In our previous study [13], microbial source tracking markers were used to identify the possible sources of faecal contamination in different water sources. Host-specific assays were used to attribute the faecal contamination to its original source. The data obtained were then used in the current study to establish the correlation between the occurrence of the target enteric pathogens and human or animal (pigs, chickens, cows, and dogs) sources of faecal contamination. Table 3 shows the data obtained from the above-mentioned study.

### 2.7. Statistical Analysis

The paired *t*-test was used to compare concentrations across assays targeting the same host, whilst the Chi-square test was used to examine the difference in positive ratios between assays targeting the same host and other hosts in the case of faecal source samples and water samples, respectively. The correlation between enteric pathogens and the different sources of contamination in water samples was analysed using a paired *t*-test and Pearson correlation coefficient ®. The results were reported to be statistically significant if the *p*-value was less than 0.05. 

## 3. Results 

### 3.1. Parameters of qPCR Assay in Detection and Quantification of Enteric Pathogens

**Figure 2:**
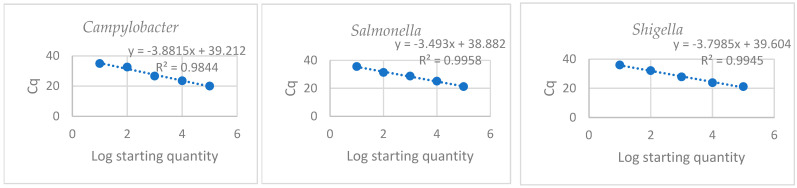
Standard curves for enteric pathogens.

Using the standard curves, the efficiency of the PCR and limit of detection (LOD) for each assay were determined. Further, the lower limit of quantification (LLOQ) and cut-off values for each assay were also determined. The efficiency of the different PCR assays was found to be 81% for *Campylobacter jejuni* subsp. *jejuni*, 83% for *Shigella flexneri*, and 93% for *Salmonella enterica* subsp. *enterica* serovar Typhimurium str. LT2. The LLOQ was determined by calculating the average Ct values from the standard curve (Figure 2) for each assay and they ranged from 27.5% to 28.58%, as shown in Table 4. The y-intercept was used as the cut-off value for each assay; any sample that amplified below the LLOQ/LOD and above the cut-off value was regarded as negative. Table 4 and Figure 2 below shows the parameters of the qPCR assays used for the detection of the target enteric pathogens. 

### 3.2. The Prevalence of Campylobacter jejuni subsp. jejuni, Shigella flexneri, and Salmonella enterica subsp. enterica Serovar Typhimurium str. LT2 in Water Sources per Season

**Figure 3:**
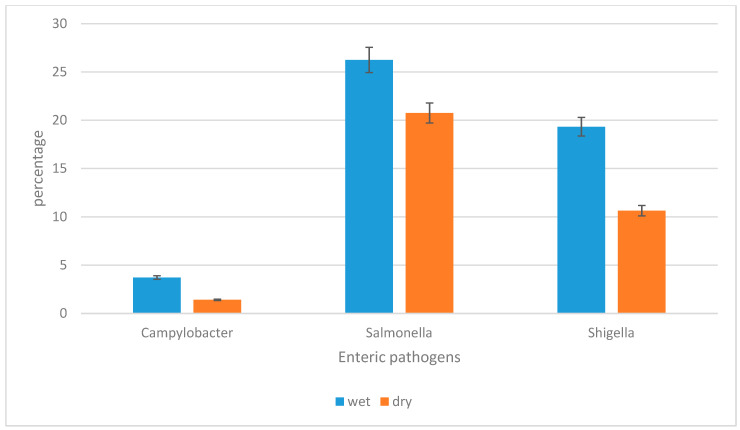
The prevalence of *Campylobacter jejuni* subsp. *jejuni, Shigella flexneri*, and *Salmonella enterica* subsp. *enterica* serovar Typhimurium str. LT2 during wet and dry seasons.

A multiplex real-time PCR assay was employed to track and detect different enteric pathogens in water sources. *Salmonella enterica* subsp. *enterica* serovar Typhimurium str. LT2 was the most prevalent enteric pathogen detected during dry and wet seasons, followed by *Shigella flexneri*, and lastly by *Campylobacter jejuni* subsp. *jejuni.* In the wet season, about 49.29% (n = 278/564) of the samples were found to be positive for either one of the target microorganisms, of which 26.24% (n = 148/564) were positive for *Salmonella enterica* subsp. *enterica* serovar Typhimurium str. LT2, 19.32% (n = 109/564) for *Shigella flexneri*, and 3.72% (n = 21/564) for *Campylobacter jejuni* subsp. *jejuni*. In the dry season, a total of 32.80% (n = 185/564) of the samples were found to be positive, of which 20.74% (n = 117/564) were positive for *Salmonella enterica* subsp. *enterica* serovar Typhimurium str. LT2, 10.63% (n = 60/564) for *Shigella flexneri*, and 1.42% (n = 8/564) for *Campylobacter jejuni* subsp. *jejuni*. The above results indicate that *Salmonella enterica* subsp. *enterica* serovar Typhimurium str. LT2 was the most prevalent enteric pathogen during both seasons and *Campylobacter jejuni* subsp. *jejuni* was the least detected pathogen during both seasons. With the exception of treatment plant final water that showed zero correlation, positive strong correlations were established between the occurrence of enteric pathogens across seasons and river water, household drinking water and treatment plant raw water (r = 0.75, r = 0.97 and r = 1, respectively). Furthermore, with the exception of household-container-stored water and treatment plant final water, the seasonal variation in the prevalence of the target enteric pathogens was statistically significant in river water (*p* = 0.05) and in treatment plant raw water (*p* = 0.01) between wet and dry seasons. Figure 3 depicts the prevalence of the different target enteric pathogens in water sources per season.

### 3.3. The Prevalence of Campylobacter jejuni subsp. jejuni, Shigella flexneri, and Salmonella enterica subsp. enterica Serovar Typhimurium str. LT2 in Water Sources per Village during Wet and Dry Seasons

**Figure 4:**
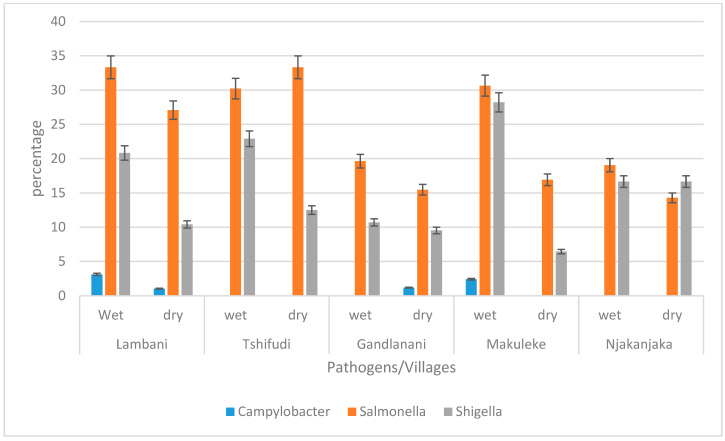
The prevalence of *Campylobacter jejuni* subsp. *jejuni*, *Shigella flexneri*, and *Salmonella enterica* subsp. *enterica* serovar Typhimurium str. LT2 in water sources used in selected villages.

The occurrence of enteric pathogens was further evaluated in all the water sources used in the selected villages for both seasons. As shown in Figure 4, in Lambani village, the most frequently identified pathogen was *Salmonella enterica* subsp. *enterica* serovar Typhimurium str. LT2 (33.33%), followed by *Shigella flexneri* (20.83%), and *Campylobacter jejuni* subsp. *jejuni* (3.13%). Furthermore, it is essential to note that the prevalence of *Campylobacter jejuni* subsp. *jejuni* was the highest in Lambani village as compared to its occurrence in other villages. In Tshifudi village, only *Salmonella enterica* subsp. *enterica* serovar Typhimurium str. LT2 and *Shigella flexneri* were detected in both seasons and the prevalence of *Salmonella enterica* subsp. *enterica* serovar Typhimurium str. LT2 was 33.33% in the dry season and 30.21% in the wet season, while for *Shigella flexneri*, it was higher (22.91%) during the wet season than during the dry season (12.5%). In Gandlanani village, during the wet season, the presence of only two pathogens was detected with *Salmonella enterica* subsp. *enterica* serovar Typhimurium str. LT2 being the most prevalent at 19.64%, followed by *Shigella flexneri* at a prevalence of 10.71%. However, during the dry season, all three target pathogens were detected, with the prevalence of *Salmonella enterica* subsp. *enterica* serovar Typhimurium str. LT2 being 15.47%, and that of *Shigella flexneri* and *Campylobacter jejuni* subsp. *jejuni* being 9.52% and 1.19%, respectively.

### 3.4. The Occurrence of Campylobacter jejuni subsp. Jejuni, Shigella flexneri, and Salmonella enterica subsp. enterica Serovar Typhimurium str. LT2 in Different Water Sources during Wet and Dry Seasons

The occurrence of enteric pathogens was found to vary with the source of water. The prevalence of enteric pathogens was found to be the highest in river water; of the 28 samples collected in the rivers during the wet season, 71.43% (n = 20/28) tested positive for *Shigella flexneri*, 60.81% (n = 17/28) for *Salmonella enterica* subsp. *enterica* serovar Typhimurium str. LT2, and 42.86% (n = 12/28) for *Campylobacter jejuni* subsp. *jejuni*. During the dry season, similar observations were noted, wherein the prevalence of target enteric pathogenic bacteria was found to be the highest in the river water as compared to other sources. However, during the dry season, *Salmonella enterica* subsp. *enterica* serovar Typhimurium str. LT2 [46.43% (n = 13/28)] was the most frequently detected pathogen, followed by *Shigella flexneri* [35.71% (n = 10/28)], while the lowest prevalence was recorded for *Campylobacter jejuni* subsp. *jejuni* [14.29% (n = 4/28)]. The difference in the occurrence of these enteric pathogens in the river water during wet and dry seasons was found to be statistically significant (*p* = 0.05), with r = 0.75, indicating a strong correlation between the two seasons (Table 5).

The presence of the selected pathogens was also detected at the point of treatment. In total, 32 samples were collected at water treatment plants prior to treatment and after treatment during the wet and dry seasons. During the wet season, 16 samples were collected in the raw water prior to treatment, and a similar prevalence of *Campylobacter jejuni* subsp. *jejuni* and *Shigella flexneri* was observed. A total of 7 (48.75%) samples were positive for both *Campylobacter jejuni* subsp. *jejuni* and *Shigella flexneri*, and 14 (87.50%) were positive for *Salmonella enterica* subsp. *enterica* serovar Typhimurium str. LT2. Furthermore, during the wet season, all 16 samples collected after chlorination (final water) tested negative for both *Campylobacter jejuni* subsp. *jejuni* and *Salmonella enterica* subsp. *enterica* serovar Typhimurium str. LT2, but two samples [2/16 (12.5%)] tested positive for *Shigella flexneri.* It is essential to note that the highest prevalence of enteric pathogens in water treatment plant samples was detected in the raw water entering the treatment plant (water before chlorination). During the dry season, the results were found to be fairly similar for the raw water samples, with *Salmonella enterica* subsp. *enterica* serovar Typhimurium str. LT2 being the most prevalent at 62.50%, and both *Campylobacter jejuni* subsp. *jejuni* and *Shigella flexneri* having a similar prevalence of 25%. However, in the final water (post-chlorination), all the samples tested negative for the target enteric pathogen during the dry season. Additionally, the seasonal variation in the occurrence of enteric pathogens in the raw water entering treatment plants was statistically significant (*p* = 0.01), showing a very strong positive correlation (r = 1). However, the seasonal variation in the occurrence of pathogens in the final water was insignificant (*p* = 0.45) and no correlation was established between the occurrence of enteric pathogens in final water and the dry and wet seasons.

At the household level, 508 water samples were tested, and the results revealed that *Salmonella enterica* subsp. *enterica* serovar Typhimurium str. LT2 (23.03%, n = 117/508)) was the most prevalent pathogen during the wet season, while *Shigella flexneri* was detected in 16.14% (82/508), and *Campylobacter jejuni* subsp. *jejuni* was the least detected at 0.39% (n = 2/508). During the dry season, the prevalence of *Salmonella enterica* subsp. *enterica* serovar Typhimurium str. LT2 and *Shigella flexneri* in household samples was 18.50% and 9.06%, respectively. *Campylobacter jejuni* subsp. *jejuni* was not detected in any household samples during the dry season. The seasonal variation in the prevalence of enteric pathogens in the household water was statistically insignificant (*p* = 0.17); however, a strong positive association (r = 0.97) was observed. Table 5 indicates the occurrence of target enteric pathogens in different water sources.

### 3.5. Correlation between Occurrence of Enteric Pathogens and Different Sources of Contamination Using Host-Specific Markers

During the study period, various activities around water sources were observed, potentially contributing to faecal contamination in water and, consequently, the occurrence of different enteric pathogens in water. Correlations between faecal contamination of water sources and host-specific Bacteroidales marker genes from our concurrent study were used to establish the relationships between pathogens and their sources [13]. Table 6 presents correlation coefficients (r) and *p*-values for various enteric pathogens during both wet and dry seasons. During the wet season, a weak negative correlation was observed between *Campylobacter jejuni* subsp. *jejuni* and all sources of contamination (ranging from −0.19 to −0.29); generally, some of these correlations were not statistically significant (*p* > 0.05), with others being significant (*p* ≤ 0.05). Positive correlations were established between *Salmonella enterica* subsp. *enterica* serovar Typhimurium str. LT2 and all the sources of contamination (ranging from 0.36 to 0.74); some of the correlations were weakly positive, such as r = 0.36, and some moderate (r = 0.55–0.62), while others showed strong correlations (r = 0.72–0.74), and these correlations were all statistically significant (*p* < 0.01). Weak positive correlations were observed between *Shigella flexneri* and some sources of contamination (ranging from 0.04 to 0.24); these correlations were statistically significant (*p* ≤ 0.05). Furthermore, in the dry season, a positive correlation was observed between *Campylobacter jejuni* subsp. *jejuni* and all sources of contamination (ranging from 0.25 to 0.84). However, most of the correlations were weak or moderate, with only one source of contamination showing a strong correlation (r = 0.84). The correlations were statistically significant (*p* < 0.03). Moderate and strong positive correlations were observed between *Salmonella enterica* subsp. *enterica* serovar Typhimurium str. LT2 and all sources of contamination (ranging from 0.45 to 0.92), and these correlations were all statistically significant (*p* < 0.01). Moreover, mixed correlations were established between *Shigella flexneri* and the sources of contamination, with some positive and some negative correlations; however, the correlations were not consistently statistically significant. In general, weaker and less significant correlations were observed during the wet season than during the dry season. In addition, a consistent positive correlation was established between *Salmonella enterica* subsp. *enterica* serovar Typhimurium str. LT2 and most sources of contamination during both the seasons. Weaker and negligible correlations between *Campylobacter jejuni* subsp. *jejuni* and contamination sources were observed during the wet season, and positive correlations in the dry season, while the correlations between *Shigella flexneri* and contamination sources appeared to be inconsistent and generally statistically insignificant. 

**Figure 5:**
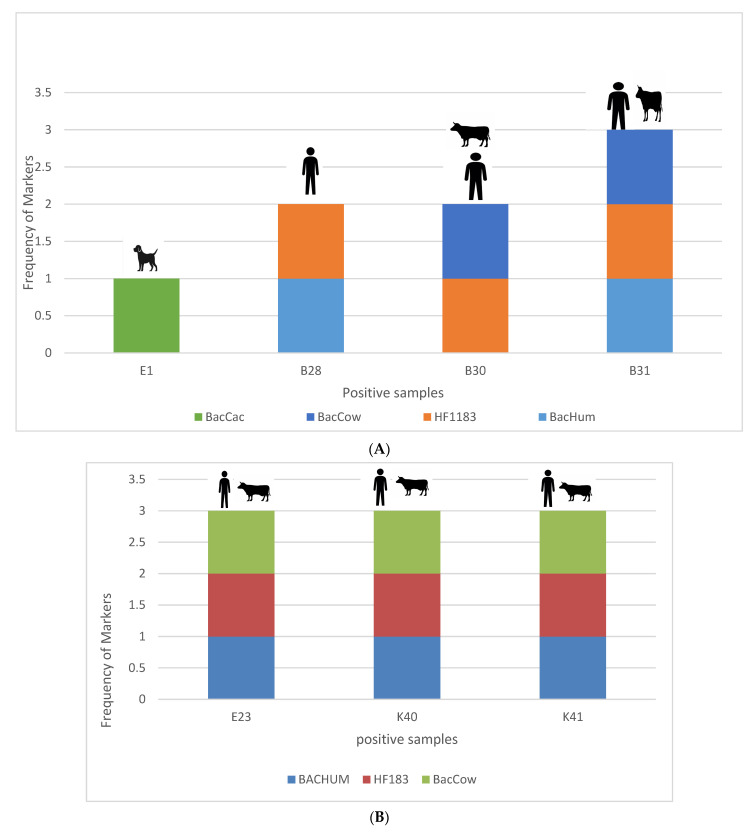

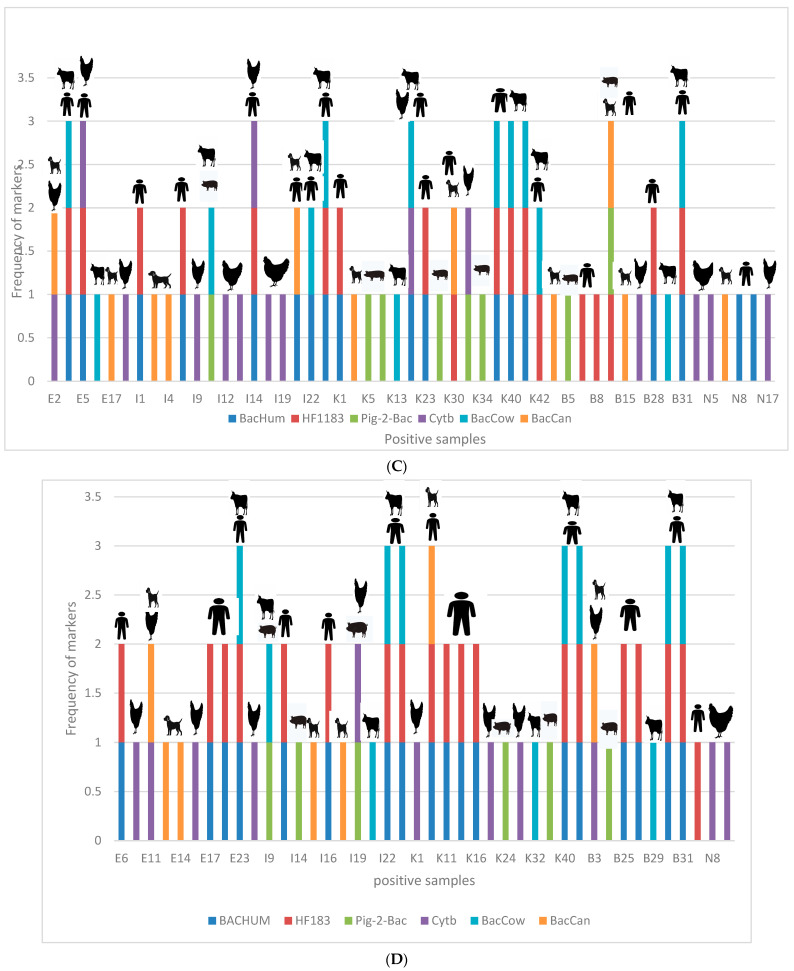

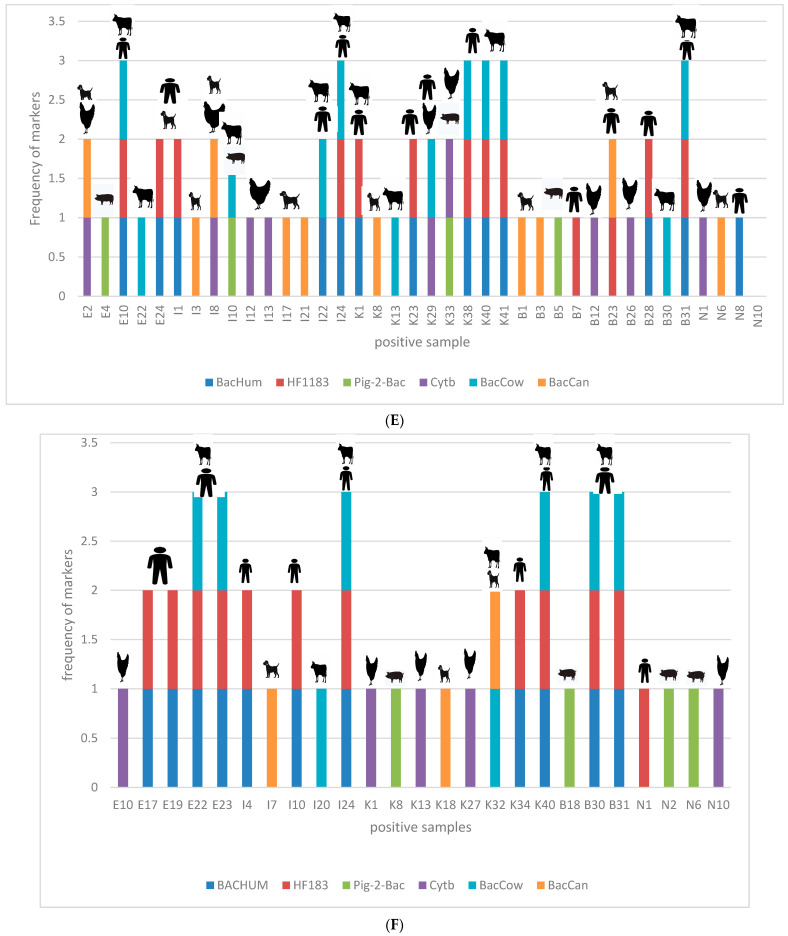
(**A**): Number of markers associated with the presence of *Campylobacter jejuni* subsp. *jejuni* in water sources during the rainy season. E1: HHS (Lambani household storage); B28: XRAW (Xikundu Water Treatment Works raw water, Xikundu); B30: ALD (Luvuvhu River downstream of Xikundu Water Treatment Works); B31: ALU (Luvuvhu River upstream of Xikundu Water Treatment Works). (**B**): Number of markers associated with the presence of *Campylobacter jejuni* subsp. *jejuni* in the water sources during the dry season. E23: XRAW (Xikundu Water Treatment Works raw water, Lambani samples); K40: KLD (Luvuvhu River downstream, Gandlanani Village); K41: KLU (Luvuvhu River upstream, Gandlanani Village). (**C**): Number of markers associated with the presence of *Salmonella enterica* subsp. *enterica* serovar Typhimurium str. LT2 in water sources during the rainy season. E2–E17: Lambani HHS; I1–I19: Tshifudi HHS; I22: XRAW: Xikundu Water Treatment Works raw water; K1–K34: Gandlanani HHS; K40–K42: Gandlanani Luvuvhu River; B5–B15: Makuleke HSS; B28: XRAW, B31: Xikundu Luvuvhu River; N5–N17: Njhakanjhaka stored borehole water. (**D**): Number of markers associated with the presence of *Salmonella enterica* subsp. *enterica* serovar Typhimurium str. LT2 in water sources during the dry season. E6–E17: Lambani HHS; E23: XRAW: Xikundu Water Treatment Works raw water; I9–I19: Tshifudi HHS, I22: XRAW; K1–K32: Gandlanani HHS; K40–K42: Gandlanani Luvuvhu River; B3–B25: Makuleke HSS, B29: XRAW; B31: Xikundu Luvuvhu River; N8: Njhakanjhaka stored borehole water. (**E**): Number of markers associated with the presence of *Shigella flexneri* in water sources during the rainy season. E2–E10: Lambani HHS; * E24: XFINAL (Xikundu Water Treatment Works final water); I3–I21: Tshifudi HHS; I24: Xikundu Luvuvhu River Extraction point; K8–K33: Gandlanani HHS; * K40: Gandlanani Luvuvhu River; B1–B26: Makuleke HSS; B30: ALD Xikundu Luvuvhu River downstream; N1–N8: Njhakanjhaka stored borehole water. (**F**): Number of markers associated with the presence of *Shigella flexneri* in possible water sources during the dry season. E10–E19: Lambani HHS; E22: Xikundu Luvuvhu River Extraction; E23: XRAW (Xikundu Water Treatment Works Raw water); I4–I20: Tshifudi HHS; I24: Xikundu Luvuvhu River; K1–K34: Gandlanani HHS; K40: Gandlanani Luvuvhu River; B18: Makuleke HSS; B30: ALD (Xikundu Luvuvhu River downstream, Makuleke Village), B31: ALU (Xikundu Luvuvhu River upstream, Makuleke village); N1–N10: Njhakanjhaka stored borehole water.

Furthermore, the association between the different enteric pathogens and host-specific markers is indicated in Figure 5A–F. As can be seen in Figure 5A, during the wet season, an association was established between the occurrence of *Campylobacter jejuni* subsp. *jejuni* in household-container-stored water and humans. Sample E1 is a sample collected in household-stored water. Dog-specific markers were also detected in the same sample, making dogs a possible source of contamination. In sample B28s, also a household stored water sample, the detected host-specific markers were human markers, suggesting that human faecal materials could be a source of contamination. Furthermore, in samples B30 and B31, both collected in the Luvuvhu River, human- and cow-specific markers were detected, which indicated that either humans or cows could be the source of faecal contamination in the river, further suggesting that the possible source of the *Campylobacter jejuni* subsp. *jejuni* detected in those samples may either be from humans or cows. During the dry season, as indicated by Figure 5B, in all the samples in which *Campylobacter jejuni* subsp. *jejuni* was detected, humans and cows were also identified as the possible sources of contamination. 

Figure 5C indicates the possible sources of *Salmonella enterica* subsp. *enterica* serovar Typhimurium str. LT2 in the different water samples during the wet season. The different host-specific markers for chickens, dogs, pigs, humans, and cows were detected in different water samples. Interestingly, in all the river water samples and treatment plant raw water samples (water before treatment), the host-specific Bacteroidales markers suggest that the source of *Salmonella enterica* subsp. *enterica* serovar Typhimurium str. LT2 in the river could either be humans or cows. The host-specific Bacteroidales markers shown in Figure 5C indicate that most of the *Salmonella enterica* subsp. *enterica* serovar Typhimurium str. LT2 in water was contributed by humans and cows, followed by chickens, dogs, and finally pigs. 

Additionally, Figure 5D shows the possible sources of contamination during the dry season. The host-specific genetic markers observed during the wet season (Figure 5C) indicated that the occurrence of *Salmonella enterica* subsp. *enterica* serovar Typhimurium str. LT2 in the river and in raw water at the point of abstraction of the water treatment plant is from humans (*BacHum* and *HF183*) and cows (*BacCow*). As indicated in Figure 5D, different markers were detected in households during the dry season. Additionally, in other water samples, multiple sources of contamination were detected in the same sample, while in other samples multiple pathogens were detected in the same sample. 

Furthermore, the occurrence of *Shigella flexneri* was also evaluated against the possible sources in both wet and dry seasons as indicated in Figure 5E,F. The results shown for the rainy season in Figure 5E suggest that the assessed sources of contamination might be the possible source of *Shigella flexneri* in the tested water samples. Interestingly, in one of the water treatment plants, in the final water (water after chlorination), *Shigella flexneri* was detected together with both the human-specific genetic markers (*BacHum* and *HF183*). These results suggest that humans are the possible source of the *Shigella flexneri* detected in the water. Since *Shigella flexneri* was detected in the treatment plant final water (post-chlorination), it was to be expected that the same pathogen would be detected in household water supplied by the treatment plant; however, in most of the households, different enteric pathogens and sources of contamination were detected other than humans. Similar results are shown in Figure 5F, which represents the findings for the dry season; different sources of contamination were identified in the same samples in which *Shigella flexneri* was detected. 

## 4. Discussion

More than 500 microorganisms can be classified as waterborne pathogens and they are responsible for waterborne diseases [17]. The burden of disease from poor water quality, sanitation, and hygiene has been established and associated with up to 4% of all deaths worldwide [18,19]. Diarrhoeal disease, for example, is known as one of the main causes of morbidity and mortality of immunocompromised individuals, especially in elderly people and children under the age of five years in developing countries. Bacterial diarrhoea in children under the age of five years has been linked to pathogens such as *Campylobacter*, *Salmonella*, and *Shigella* [20]. To protect and promote public health, strategies have been developed for managing water quality. Among these strategies, microbial source tracking (MST) plays a major role in discriminating between human and animal sources of faecal pollution to improve water quality management [21]. The main focus of this study was, therefore, tracking and detecting enteric pathogens such as *Salmonella enterica* subsp. *enterica* serovar Typhimurium str. LT2, *Shigella flexneri*, and *Campylobacter jejuni* subsp. *jejuni*. The study further established the correlation between these pathogens and the sources of faecal pollution with reference to five selected water-stressed villages of the Vhembe District Municipality. 

In this study, qPCR assays were evaluated using standard curves as shown in Figure 2, with efficiencies ranging from 81% to 93%. The acceptable efficiency for a successful qPCR assay is between 80% and 110% [22]. The presence of target enteric pathogens in water sources of rural communities was confirmed, raising concerns about potential waterborne diseases. *Salmonella enterica* subsp. *enterica serovar* Typhimurium str. LT2 was the most prevalent pathogen, except in river water during the wet season, where *Shigella flexneri* predominated (Table 5 and Figure 3). *Campylobacter jejuni* subsp. *jejuni* was the least detected in river and treated water, while it could not be detected in household-container-stored water during the dry season. Positive strong correlations were observed between pathogen occurrence across seasons in river, household drinking, and treatment plant raw water (Table 5). Seasonal variation in pathogen prevalence was statistically significant in river and treatment plant raw water. These results align with previous studies showing *Salmonella* spp. as the most frequently detected pathogen in water sources, often linked to faecal contamination and discharge of untreated waste [5,23,24]. Anthropogenic activities such as swimming, bathing, and washing were observed along the river, likely contributing to faecal pollution and the presence of enteric pathogens [25]. *Shigella*, among other harmful bacteria, was frequently detected in water samples. The diversity of enteric pathogens found in drinking water may vary by region and be influenced by seasonal fluctuations and anthropogenic activities near water sources [23,26,27,28]. In the current study, the prevalence of the target pathogens was higher during the wet season as compared to the dry season (Figure 3), and the results are consistent with the findings of prior research [26,27,29]. Heavy rainfall events during the wet season can cause surface runoff containing various contaminants to enter the water sources, resulting in an expanded microbial population in water. Cooler temperatures may also enhance the growth of many enteric pathogens, increasing the concentration of bacteria in water. However, during the dry season, bacterial cells are susceptible to desiccation and may survive but cannot grow and divide, and thus the concentration of bacteria may be lower. Because of the aforementioned factors, the high incidence of enteric infections during the wet season relative to the dry season is not surprising.

Furthermore, the occurrence of these enteric pathogens was evaluated at different sampling points (water treatment plants, rivers, and households). At the water treatment plants, the target pathogens were frequently detected in the raw water (before chlorination); however, *Shigella flexneri* was detected in two samples of the treatment plant final water (Table 5). Chlorination is the disinfection method commonly used worldwide, especially in developing countries. However, previous studies have demonstrated that chlorination as a cost-effective water treatment process cannot be a guarantee to safeguard against bacteria in treated water [30,31]. This clearly explains the detection of some enteric pathogens in the final water produced by the water treatment plant, in spite of a decrease in their concentration (Table 5). The detection of pathogens at the point of treatment implies that a pathogen detected at the treatment plant level has a high probability of being detected at the household level. Water contamination in households may also occur as a result of poor hygiene, failure to cover storage containers, ways of dispensing water, and animals roaming freely in the house. The findings of this investigation are in agreement with those of a study conducted in 2022 by Khabo-Mmekoa and colleagues [32], who found that household water samples were mainly contaminated by enteric pathogens such as *Salmonella* and *Shigella*. Muringani et al. [33] also reported similar findings, showing that 87.5% of all water samples analysed from both treated and untreated sources in their study were contaminated with pathogenic bacteria. The findings of this study will assist water service authorities in rural areas to develop strategies aimed at reducing the exposure of humans to pathogens that cause diseases, and to create an awareness that household factors such as hygiene practices might be responsible for the recontamination of treated water.

Environmental contamination exposes water sources to a constant risk and threat; contamination from either point or non-point sources has made water sources susceptible to pathogenic bacteria and viruses that threaten the health of humans. Consequently, it is crucial to establish the association between the sources of faecal contamination and the prevalence of target pathogens in water. As mentioned above, the current study was conducted simultaneously with one that focused on the identification of predominant faecal contamination sources in similar water sources using host-specific genetic markers [13]. Considering the results obtained, a correlation was established between pathogens detected in water sources and faecal contamination sources. The results showed that human or animal faecal matter present in water is contributing to the presence of enteric pathogens in water. Using microbial source tracking markers, the different enteric pathogens isolated in each of the water samples were correlated to their possible source. As reported in Table 6, mixed correlations between *Shigella flexneri* and the sources of contamination were observed, with some negligible positive and some negative correlations. During the wet season, weak negative and weak correlations (r = −0.2 to r = 0.24) were found, while the dry season showed varying correlations (negligible negative to moderate positive) without consistent statistical significance. *Salmonella enterica* consistently correlated positively with contamination sources in both seasons (r = 0.34 to r = 0.92). *Campylobacter* had weaker correlations in the wet season and positive correlations in the dry season, except for specific cases (*p* ≤ 0.05). Furthermore, the target enteric pathogens were associated with the different sources of contamination in water. *Campylobacter jejuni* subsp. *jejuni* was frequently isolated from river water, and the presence of dog markers (*BacCan*) suggested that dog faeces might be a possible source of contamination. Dogs can transmit a variety of bacterial infections to people and serve as a key reservoir for zoonotic diseases [34,35,36,37]. Further, *Campylobacter jejuni* subsp. *jejuni* was found in river water, alongside human- and cow-specific Bacteroidales genetic markers, suggesting humans and cows as possible sources. The human-associated genetic marker *BacHum-UCD* has also been linked to *Campylobacter* [38,39]. While poultry is commonly associated with *Campylobacter*, Wilon et al. [40] revealed that ruminants are the second highest source of *C. jejuni* in humans.

Additionally, in the same water samples collected during the study period, *Salmonella enterica* subsp. *enterica* serovar Typhimurium str. LT2 and *Shigella flexneri* were detected and their possible sources of contamination were identified (Figure 5A–F). The possible sources of all the pathogens detected in the river water were either humans or cows. Savichtcheva et al. [41] reported that total and human-specific *Bacteroides* 16S rRNA genetic markers showed a significant correlation with *Salmonella* and *Shigella.* These markers owe their presence in the water samples to the faecal matter and various anthropogenic activities observed in the vicinity of the river.

In household water samples, varying host-specific markers believed to be the possible sources of enteric pathogens were detected. *Salmonella enterica* subsp. *enterica* serovar Typhimurium str. LT2 contamination in domestic water samples has been linked to poultry, dogs, pigs, cows, and even humans. Human-specific markers were shown to be the most common source of *Salmonella enterica* subsp. *enterica* serovar Typhimurium str. LT2, followed by the chicken-associated marker *Cytb* (Figure 5C,D). According to Mughini-Gras et al. [42], pigs and cattle were the most likely reservoirs of salmonellosis in children, while hens were linked to salmonellosis in adults. *Salmonella* is naturally found in both wild and domestic animals; however, it is most typically detected in the intestines of chickens [43]. Another study found that dogs are important asymptomatic reservoirs of potentially dangerous *Salmonella* strains [44]. In 2020, Askari and co-workers [45] reported the possible transfer of antibiotic-resistant *Salmonella* from dogs to humans. As can be seen from the earlier research, there is strong evidence that the faeces of these animals may likely be associated with the occurrence of *Salmonella*.

Similarly, several sources of contamination were identified in the water samples in which *Shigella flexneri* was detected (Figure 5E,F)*. Shigella flexneri* was detected in samples of the final water of the water treatment plant, at the same time as human-specific Bacteroidales genetic markers. It was to be expected that the same pathogen would be detected in household water supplied by the treatment plant; however, in most of the households, different enteric pathogens and sources of contamination were detected other than humans, suggesting that the supplied water is also being polluted at the household level. According to the findings of the current investigation, human-specific genetic markers were commonly detected as a likely source of *Shigella flexneri* in water. As stated by Shi et al. [44], humans and other primates are natural hosts of *Shigella*. However, new hosts, such as calves and piglets, have emerged over time. In addition, these authors have pointed out that chickens are also *Shigella* hosts, and a probable cross-infection between poultry and humans has been observed [46]. It is important to highlight that other sources of contamination were observed in other water samples, making it difficult to adequately link the contamination to its exact source. The current study only focused on the detection of three enteric pathogens; therefore, it is not surprising that sources of contamination were detected in water samples that tested negative for either of the enteric pathogens. If enteric pathogens were detected in a sample without a possible source, a source other than the ones tested for could be a possible source of contamination in the water sample.

## 5. Conclusions

The data acquired in this study clearly show that activities occurring in and around water sources significantly contribute to faecal pollution of water sources. The presence of enteric pathogens in water demonstrates the importance of raising awareness about water treatment. The enteric pathogens were also detected in household drinking water, implying that people living in rural areas are at a greater risk of waterborne illnesses. The findings of this study indicate the need for additional intervention in water purification at both the household and water treatment plant levels. Knowledge of the source of contamination will also aid in the prevention of future contamination concerns by implementing strategies that can adequately counteract the sources of faecal contamination with the aim of improving water quality management. Good quality water will not only reduce the frequency of waterborne infections, but will also help to improve people’s lives, particularly in rural communities. Future studies should be conducted to gain a better understanding of the transmission of enteric pathogens from the primary source of water to the point of use. Further research into the relationship between enteric pathogens and microbial source tracking markers cannot be underestimated.

## 6. Study Limitations

This study focused on detecting three specific enteric pathogens (*Campylobacter jejuni* subsp. *jejuni*, *Salmonella enterica* subsp. *enterica serovar* Typhimurium str. LT2, and *Shigella flexneri*). Other potential pathogens or sources of contamination may not have been examined, limiting the comprehensive understanding of water quality. While the study attempted to identify potential contamination sources through host-specific genetic markers, accurately attributing pathogens to specific sources can be challenging due to multiple potential sources in the environment. To successfully correlate the occurrence of enteric pathogens with a source of contamination, the pathogens need to be tracked back to one original source. The sensitivity of the detection methods used in the study may influence pathogen identification. Some pathogens may exist in low concentrations, leading to potential underestimation of their presence.

## Figures and Tables

**Table 1:** Total number of water samples collected from the main sources to the point of use during both dry and wet seasons.

Sources of Water	Villages					
	Lambani	Tshifudi	Gandlanani	Makuleke	Njhakanjhaka	Total
WPPB	0	0	0	0	112	112
River	16	16	16	16	0	64
Treatment plant	16	16	16	16	0	64
Household storage	24	8	152	72	48	304
Household/communal tap	136	160	144	144	0	584
Total	192	200	328	248	160	1128

WPPB: water purchased from private boreholes.

**Table 2:** Primers and probes used for the detection of enteric pathogens.

Pathogen	Target Gene	Primer/Probe	Sequence 5–3	Conditions	Reference
*Campylobacter jejuni* subsp. *jejuni*	*gyrA*	366F	CTA TAA CAA CTG CAC CTA CTA AT	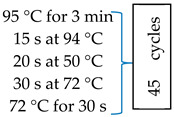	Wiemer et al. (2011) [16]
		614R	ATG AAA TTT TTG CCA GTG GTG		
		409P	Fam-CTT AAT AGC CGT CAC CCC AC-Tam.		
*Shigella flexneri*	*ipaH*	1635F	CAG AAG AGC AGA AGT ATG AG		
		1804R	CAG TAC CTC GTC AGT CAG		
		1747P	ROX-ACA GGT GAT GCG TGA GAC TG-BHQ2		
*Salmonella enterica* subsp. *enterica* serovar Typhimurium str. LT2	*ttrC*	4136F	AAT TAG CCA TGT TGT AAT CTC		
		4315R	ATT GTT GAT TCA GGT ACA AAC		
		4163P	JOE-CAA GTT CAA CGC GCA ATT TA-BHQ1a		

**Table 3:** The prevalence of sources of faecal contamination in water sources.

Sources of Contamination	Water Source Categories							
	Wet Season							
	Household		River		Treatment Plant		Total	
	Frequency	Percent	Frequency	Percent	Frequency	Percent	Frequency	Percent
*BacHum*	20	10.86	3	18.75	5	35.71	28	13.08
*HF183*	23	12.5	7	43.75	5	35.71	35	16.35
*Cytb*	44	23.91	0	0	0	0	44	20.56
*BacCow*	26	14.13	5	31.25	4	28.57	35	16.35
*BacCan*	41	22.28	0	0	0	0	41	19.15
*Pig-2-Bac*	30	16.3	1	6.25	0	0	31	14.48
Total	184	100	16	100	14	100	214	100
	Dry Season							
*BacHum*	34	23.94	3	21.43	3	18.75	40	23.26
*HF183*	31	21.83	3	21.43	2	12.5	26	20.93
*Cytb*	23	16.2	1	7.14	2	12.5	26	15.12
*BacCow*	13	9.15	5	35.71	5	31.25	23	13.37
*BacCan*	21	14.79	2	14.29	2	12.5	25	14.53
*Pig-2-Bac*	20	14.08	0	0	2	12.5	22	12.79
Total	142	100	14	100	16	100	172	100

*BacHum*: Human; *HF183*: Human; *Cytb*: Chickens; *BacCow*: Cows; *BacCan*: Dogs; *Pig-2-Bac*: pigs.

**Table 4:** Standard curve parameters for enteric pathogens.

Standard Curve Parameters for Enteric Pathogens						
Target	Slope	y-Intercept	Efficiency	LLOQ (Ct)	Gene Copies per μL	Log10 Gene Copies per ng
*Campylobacter jejuni* subsp. *jejuni*	−3.882	39.212	81	27.5	4.00 × 10^37^	37.60
*Salmonella enterica* subsp. *enterica* serovar Typhimurium str. LT2	−3.493	38.882	93	28.39	3.32 × 10^39^	39.52
*Shigella flexneri*	−3.799	39.604	83	28.58	1.01 × 10^39^	39.00

**Table 5:** The occurrence of enteric pathogens in different water sources.

		Enteric Pathogens							
Sources of Water		*Campylobacter jejuni* subsp. *jejuni*		*Salmonella enterica* subsp. *enterica* serovar Typhimurium str. LT2		*Shigella flexneri*		*p*-Value	Pearson Correlation (r)
River n = 28 per season	WET	12	42.86	17	60.71	20	71.43	0.05	0.75
	DRY	4	14.29	13	46.43	10	35.71		
Treatment plant (RW) n = 16 per season	WET	7	43.75	14	87.5	7	43.75	0.01	1
	DRY	4	25	10	62.5	4	25		
Treatment plant (FW) n = 16 per season	WET	0	0	0	0	2	12.5	0.42	0
	DRY	0	0.00	0	0	0	0.00		
Household (drinking water) n = 508 per season	WET	2	0.39	117	23.03	82	16.14	0.17	0.97
	DRY	0	0	94	18.50	46	9.06		

n: total number of samples tested. RW: raw water (water before treatment). FW: final water (water after treatment).

**Table 6:** Correlation between target enteric pathogens and the different sources of contamination.

	Enteric Pathogens (Correlation Coefficient (r))					
Sources of Contamination	WET SEASON					
	*Campylobacter jejuni* subsp. *jejuni*		*Salmonella enterica* subsp. *enterica* Serovar Typhimurium str. LT2		*Shigella flexneri*	
	r	*p*	r	*p*	r	*p*
*BacHum*	−0.19	0.05	0.6	0.01	0.05	0.01
*HF183*	−0.25	0.08	0.55	0.01	0.16	0.01
*Cytb*	−0.08	0.01	0.72	0.01	0.24	0.02
*Pig-2-Bac*	−0.01	0.01	0.62	0.01	0.04	0.01
*BacCow*	−0.29	0.2	0.36	0.01	−0.2	0.03
*BacCan*	−0.02	0.02	0.74	0.01	0.2	0.02
	DRY SEASON					
	*Campylobacter jejuni* subsp. *jejuni*		*Salmonella enterica* subsp. *enterica* serovar Typhimurium str. LT2		*Shigella flexneri*	
	r	*p*	r	*p*	r	*p*
*BacHum*	0.34	0.03	0.92	0	0.08	0.3
*HF183*	0.46	0.02	0.92	0	0.25	0.13
*Cytb*	0.65	0.02	0.45	0.01	0.54	0
*Pig-2-Bac*	0.25	0.02	0.78	0.01	0.25	0.01
*BacCow*	−0.1	0.09	0.78	0.01	−0.05	0.1
*BacCan*	0.84	0.02	0.66	0.01	0.5	0.01

## Data Availability

All the relevant data are included in the article.
